# Pathogenicity of *Tolypocladium* spp. Against *Plutella xylostella*: Effects on Immune Enzyme Activities and Gene Expression Profile

**DOI:** 10.3390/insects16080859

**Published:** 2025-08-18

**Authors:** Ni Cai, Zhigang Zhang, Babar Hussain Chang, Zhijun Qiao, Fang Liu, Xiangqun Nong, Kaimei Wang

**Affiliations:** 1 Hubei Biopesticide Engineering Research Centre, Hubei Academy of Agricultural Sciences, Wuhan 430064, Chinaq1158007294@163.com (Z.Q.);; 2 Department of Plant Protection, Sub-Campus Umerkot, Sindh Agriculture University, Tandojam 7600, Pakistan; babar_chang@yahoo.com; 3 School of Life Science and Technology, Wuhan Polytechnic University, Wuhan 430030, China; 4 State Key Laboratory for Biology of Plant Diseases and Insect Pests, Institute of Plant Protection, Chinese Academy of Agricultural Sciences, Beijing 100193, China

**Keywords:** *Tolypocladium*, *Plutella xylostella*, Toll pathway, enzyme activity, virulence

## Abstract

**Simple Summary:**

*Tolypocladium* fungi are recognized for producing valuable bioactive compounds and infecting insects, making them promising agents for biological pest control. *Plutella xylostella* (diamondback moth) is a major pest of cruciferous crops worldwide, with widespread resistance to chemical insecticides. In this study, four *Tolypocladium* strains (three *T. inflatum* and one *T. cylindrosporum*) were isolated from soil and evaluated against *P. xylostella*. Three strains (O1, O3, and N8) caused over 90% larval mortality within short lethal times (LT_50_: 3.89–4.45 days). Strain N8 exhibited notable heat tolerance, enhancing its suitability for field applications. The fungi suppressed the pest’s immune system by modulating Toll pathway gene expression and altering key enzyme activities. Immune enzymes (PO, CAT, POD) were initially activated but later inhibited, while detoxification enzymes (GSTs, CarE, AChE) were largely suppressed, weakening the insect’s defense against infection. These findings highlight *T. inflatum* O1 and *T. cylindrosporum* N8 as effective, eco-friendly biocontrol candidates for sustainable *P. xylostella* management, offering a potential alternative to chemical pesticides and helping mitigate resistance development.

**Abstract:**

(1) Background: *Tolypocladium* spp. are fungi known for producing cyclosporin A and their ability to infect insects. However, their pathogenicity against the lepidopteran pest *Plutella xylostella* has not been previously reported. (2) Methods: Four *Tolypocladium* strains were isolated from soil and identified through morphological and phylogenetic analyses (ITS, gene sequencing). Growth rates, sporulation capacity, and stress tolerance (45 °C heat, UV) were evaluated. Pathogenicity was assessed via larval bioassays, and immune responses were analyzed by quantifying Toll pathway gene expression and enzyme activities (PO, CAT, POD, GSTs, CarE, AChE) from 24 to 96 h post-inoculation. (3) Results: Strains N8-SF-04092 and O1/O2/O3-SF-04630/04927/04931 were identified as *Tolypocladium cylindrosporum* and *Tolypocladium inflatum*, respectively. Strain O2 showed the highest growth rate (*p* < 0.05), while O3 and N8 exhibited superior sporulation (>7 × 10^5^ spores/mm^2^). N8 also demonstrated notable thermotolerance. In pathogenicity assays, O1, O3, and N8 caused 98.3%, 93.3%, and 96.7% larval mortality, respectively, with LT_50_ values (3.89–4.45 days) significantly lower than O2 (*p* < 0.05). Immune gene expression in *P. xylostella* was transiently activated at 24 h but suppressed from 48 to 96 h by N8 (*p* < 0.05), while O1 induced partial activation at 24 h and 96 h but suppression at 48 h and 72 h. Protective enzymes (PO, CAT) were initially upregulated (24–48 h) but inhibited after 72 h (*p* < 0.01). POD activity showed opposing trends between O1 (initially activated then suppressed) and N8 (initially suppressed then activated). Detoxification enzymes (GSTs, CarE, AchE) were predominantly suppressed, except for GSTs, which increased at 72–96 h. (4) Conclusions: Strains O1 and N8 exhibit high virulence against *P. xylostella* by disrupting immune responses through dynamic modulation of Toll pathway genes and enzyme activities. The thermotolerance of strain N8 further enhances its promising biocontrol agent for field application.

## 1. Introduction

The genus *Tolypocladium* was first established by W. Gams in 1971 to accommodate three novel fungal species isolated from soil: *Tolypocladium cylindrosporum*, *Tolypocladium geodes*, and *Tolypocladium inflatum*. Since then, additional species such as *Tolypocladium lignicola*, *Tolypocladium parasiticum*, and *Tolypocladium trigonosporum* have been added, expanding the genus’s ecological and biological diversity [1,2,3,4]. In 1994, *T. inflatum* was formally designated as the type species of the genus due to its ability to produce cyclosporin A and its increasing importance in research [5,6].

*Tolypocladium* species are known to exhibit dual roles as both saprophytes and entomopathogens [7]. Among them, *T. cylindrosporum* (Ascomycota: Hypocreales) is a well-characterized insect pathogen and has been evaluated for its potential in biological control programs [8,9]. It infects a wide range of insect hosts across various orders, including Coleoptera (Elateridae), Lepidoptera (Noctuidae), Diptera (Culicidae, Bibionidae, Anthomyiidae), and Hymenoptera (Sericidae, Formicidae), and is also commonly isolated from soil environments [10]. Notably, its larvicidal activity against mosquito vectors such as *Aedes*, *Anopheles*, and *Culex* has been demonstrated in several studies [11,12,13]. In contrast, *T. inflatum* is primarily known for its pharmaceutical application as the producer of cyclosporin A, a widely used immunosuppressant in transplant medicine and autoimmune therapies [14,15]. Despite its clinical significance, the entomopathogenic potential of *T. inflatum* remains poorly understood, with limited investigations into its pathogenicity against insect hosts.

The diamondback moth (*Plutella xylostella* L.), a lepidopteran pest in the family Plutellidae, is among the most globally destructive insects affecting cruciferous crops [16,17,18]. Originally native to the Mediterranean region, it has now achieved a nearly cosmopolitan distribution due to its strong migratory capacity and adaptability to diverse climates [19]. Cruciferous vegetables, characterized by rapid growth and high market demand, are cultivated year-round in many regions. This continuous crop availability supports persistent population growth and infestation cycles of *P. xylostella* [20]. Since the 1950s, the extensive use of broad-spectrum insecticides has disrupted natural enemy populations and driven the evolution of insecticide resistance in *P. xylostella*, greatly complicating pest control efforts. As a result, there is a pressing need to develop sustainable and environmentally friendly alternatives. Entomopathogenic fungi offer such a solution, combining low toxicity to humans and non-target organisms with the ability to cause natural epizootics in pest populations. These characteristics align well with integrated pest management (IPM) principles and sustainable agricultural goals. Several entomopathogenic fungal genera, including *Beauveria*, *Metarhizium*, and *Isaria*, have demonstrated promising efficacy against *P. xylostella*. However, the entomopathogenic potential of *Tolypocladium* spp. against this pest remains largely unexplored.

The infection process of entomopathogenic fungi typically follows a multi-step progression involving spore adhesion, germination, cuticular penetration, and internal proliferation. In response, the insect host mounts complex defense mechanisms comprising cellular and humoral immunity, as well as protective and detoxification enzymatic responses [21,22]. The humoral immune system in insects is regulated by key signaling pathways: Toll, IMD (Immune Deficiency), JAK/STAT, and JNK. Of these, the Toll pathway plays a central role in microbial recognition and antimicrobial peptide production [23,24]. Upon pathogen detection, pattern recognition receptors (PRRs) such as *GNBPs* and *PGRPs* trigger downstream signaling cascades involving *Spaetzle* activation and translocation of transcription factors like *Dorsal* and *Dif*, leading to AMP gene expression. In parallel, the insect stress response includes the induction of antioxidant and detoxification enzymes to mitigate damage from infection and maintain physiological balance [25]. Key antioxidant enzymes such as catalase (CAT), peroxidase (POD), and phenoloxidase (PO) combat oxidative stress, while detoxification enzymes, including glutathione S-transferases (GSTs), carboxylesterases (CarE), and acetylcholinesterase (AChE), help neutralize and eliminate toxic substances [26,27]. In this study, four *Tolypocladium* strains were isolated from soil and identified based on morphological and molecular markers. Their growth, sporulation capacity, and tolerance to environmental stressors (heat and UV) were systematically evaluated. In addition, their pathogenicity against *P. xylostella* was assessed, along with the temporal expression patterns of Toll pathway immune-related genes and changes in enzymatic activities following infection. This study provides foundational insights into the entomopathogenic potential of *Tolypocladium* spp., supporting future research and development of novel fungal biocontrol agents targeting lepidopteran pests.

## 2. Materials and Methods

### 2.1. Biomaterials and Their Culture Conditions

Four *Tolypocladium* strains (N8-SF-04092, O1-SF-04630, O2-SF-04927, and O3-SF-04931) previously isolated from soil samples were obtained from our laboratory culture collection. For spore production, each strain was cultured on potato-sucrose agar plates (PSA), composed of 200 g potato infusion, 20 g sucrose, 5 g yeast extract, and 20 g agar per liter, with the medium’s natural pH. Inoculated plates were incubated at 28 °C for 10 days to promote sporulation. Spores were harvested for use in subsequent experiments.

The *P. xylostella* test population was maintained in an insectary on a commercial artificial diet (Southland Products, Inc.—Entomological Solutions (Lake Village, AR, USA), Catalog No. 080222DBM) under controlled environmental conditions: 25 ± 1 °C, 50 ± 5% relative humidity, and a 16:8 h light/dark photoperiod. All bioassays and enzyme activity analyses were performed using third-instar larvae from this colony.

### 2.2. Molecular Identification of Fungal Strains

The *Tolypocladium* strains were cultured on potato-sucrose agar (PSA) plates overlaid with sterile cellophane membranes and incubated at 28 °C for 5–7 days to obtain sufficient mycelial biomass. Genomic DNA was extracted using a commercial fungal DNA extraction kit (Jianshi Biotechnology, Beijing, China) following the manufacturer’s protocol. The internal transcribed spacer (ITS) region of rDNA was amplified using universal fungal primers ITS1 (5′-TCCGTAGGTGAACCTGCGG-3′) and ITS4 (5′-TCCTCCGCTTATTGATATGC-3′) [28]. PCR amplification conditions were as follows: initial denaturation: 94 °C for 3 min; 33 cycles of 94 °C for 30 s, 55 °C for 30 s, and 72 °C for 1 min; and a final extension at 72 °C for 10 min. PCR products were purified and sequenced commercially (Tsingke Biotechnology Co., Ltd., Beijing, China). The resulting ITS sequences were subjected to BLASTn analysis against the NCBI nucleotide database. Molecular identification was combined through the integration of BLAST results with morphological observations.

### 2.3. Phylogenetic Analysis and Multiple Sequence Alignment

The obtained ITS sequences were submitted to Genebank (O1-SF-04630 accession No. PV997985, O2-SF-04927 accession No. PV997986, O3-SF-04931 accession No. PV997987, N8-SF-04092 accession No. PV997984) and aligned with reference sequences of *Tolypocladium* species retrieved from GenBank using BioEdit Sequence Alignment Editor (v7.2.5). Sequence alignments were saved in FASTA format and used for phylogenetic analysis. Phylogenetic analysis was performed using the neighbor-joining (NJ) method in MEGA X with 1000 bootstrap replicates. Final taxonomic classification of each strain was determined on a combined analysis of morphological characteristics and phylogenetic relationships. Bootstrap support values > 50% were considered statistically significant; values below 50% were omitted for clarity.

### 2.4. Evaluation of Fungal Growth and Sporulation Capacity

Conidial suspension of each *Tolypocladium* strain was prepared in sterile distilled water containing 0.05% Tween-80 to a final concentration of 1.0 × 10^7^ spores/mL. A 10 µL aliquot of each suspension was spot-inoculated onto sterile PSA plates using 5 mm diameter filter paper discs placed at the center. Plates were incubated at 28 °C in an inverted position, with three replicates per strain. Colony diameters were measured on days 3 and 7 post-inoculation using a digital caliper. Measurements were taken in two perpendicular directions for each colony, and the corrected growth rate was calculated as the difference between 7-day and 3-day diameters. After 8 days of incubation, colony morphology, including margin, pigmentation, and texture, was visually assessed. Conidial morphology was examined microscopically. For sporulation quantification, four 5 mm mycelial plugs were collected from the mid-radius region of each colony and vortexed in 0.1% Tween-80 solution to release conidia. Spore concentration was determined using a hemocytometer and expressed as sporulation density (spores/mm^2^), with three technical replicates per strain.

### 2.5. Environmental Stress Tolerance Assays

#### 2.5.1. Thermotolerance Assay

An aliquot (100 µL) of *Tolypocladium* sp. conidial suspension (1.0 × 10^7^ spores/mL) was subjected to heat shock at 45 °C in a metal bath for 0, 10, 30, 60, 120, and 300 min. After treatment, 30 µL of the suspension was evenly spread on germination medium (containing per liter: 1 g glucose, 0.5 g peptone, and 20 g agar) and incubated at 28 °C for 18 h. Subsequently, 1 cm^2^ agar blocks were excised from each plate and mounted on a microscope slide. Spore germination was assessed under an optical microscope by randomly examining 100 conidia per sample. A spore was considered germinated if its germ tube length exceeded 50% of the spore diameter. Each treatment was performed in triplicate. Germination rate was calculated as Germination rate = (Number of germinated spores/Total number of spores) × 100.

#### 2.5.2. UV Resistance Assay

A 30 µL aliquot of conidial suspension (1.0 × 10^7^ spores/mL) was evenly spread on germination medium (as previously described in Section 2.5.1). Petri dish lids were removed, and plates were exposed to UV radiation (290 nm, 847.90 mWm^−2^) for 0 (control), 1, 3, and 6 h. Plates were then immediately covered and incubated in an inverted position at 28 °C for 18 h. Germination rate was determined as previously described. Each treatment was performed with three biological replicates.

### 2.6. Virulence Bioassays Against P. xylostella

The virulence of *Tolypocladium* spp. against third-instar larvae of *P. xylostella* was evaluated using a diet-incorporation bioassay. Artificial diet (1 mL per well) was dispensed into 24-well culture plates. A conidial suspension (1 × 10^8^ spores/mL in 0.05% Tween-80) was prepared and verified by hemocytometer count. Each well received 80 µL of the suspension, which was evenly distributed by gentle shaking. After 2 h of air-drying at room temperature, five larvae were placed in each well. Four wells constituted one biological replicate, with three replicates per treatment. Control group received 80 µL of sterile 0.05% Tween-80. Insects were observed daily for 7 days, and mortality was recorded based on the lack of movement upon gentle prodding with a fine brush. Dead larvae were removed promptly. Cumulative mortality rates were analyzed using column charts. Mortality data were corrected using Abbott’s formula when control mortality exceeded 10%.

Cumulative mortality (%) = (Number of dead individuals at time T/Total initial number) × 100

The initial conidia suspension (1 × 10^8^ spores/mL) was serially diluted to produce five logarithmic concentrations ranging from 1 × 10^7^ to 1 × 10^4^ spores/mL. Bioassays were performed as previously described. Time–dose–mortality data were analyzed using a three-parameter TDM (time–dose–mortality) model [29], which simultaneously estimates both dose–response and time-effect parameters through a regression equation. The median lethal times and ninety lethal times (LT_50_ and LT_90_, at specific doses) and median lethal concentration and ninety lethal concentrations (LC_50_ and LC_90_, at day 7) were calculated, with 95% fiducial limits derived from probit analysis.

### 2.7. Determination of Protective and Detoxification Enzyme Activities in P. xylostella

#### 2.7.1. Sample Preparation

Larvae of *P. xylostella* were treated with a conidial suspension (1.0 × 10^8^ spores/mL in 0.05% Tween-80), while the control group received sterile 0.05% Tween-80. The diet-incorporation method was applied in 24-well plates (≥10 third-instar larvae per well; 24 wells total for one biological replicate, grouped into six sets with three biological replicates each). Samples were collected at 24 h, 48 h, 72 h, and 96 h post-treatment. For each replicate, 2–3 larvae were pooled, and a total of 50 larvae were used per assay sample. Samples were flash-frozen in liquid nitrogen and stored at −80 °C.

#### 2.7.2. Enzyme Activity Assays

A total of 150 *P. xylostella* larvae were homogenized in liquid nitrogen, and homogenate was divided into three subsamples (0.1 g each) for enzymatic analysis and RNA extraction. The activities of protective enzymes, phenoloxidase (PO), catalase (CAT), and peroxidase (POD), as well as detoxification enzymes glutathione S-transferases (GSTs), acetylcholinesterase (AChE), and carboxylesterase (CarE) were quantified using commercially available assay kits (Jiangsu Aidissheng Biological Technology Co., Ltd., Yancheng, China). All procedures were conducted in accordance with manufacturer’s instructions, with three technical replicates per sample. Absorbance reading was obtained using VersaMax microplate reader (Molecular Devices, Shanghai, China). Enzyme activities were calculated based on kit-specific formulas and expressed in international units (U), defined as the amount of enzyme required to produce change in absorbance of 1.0 at the specified wavelength per minute per gram of fresh tissue under optimal assay conditions.

### 2.8. Analysis of Immune Signaling Pathway-Related Gene Expression in P. xylostella

Total RNA was extracted from 0.1 g of homogenized *P. xylostella* larvae using TRIzol^®^ Reagent (Invitrogen, Waltham, MA, USA) following the manufacturer’s protocol. RNA concentration and purity were assessed using a NanoPhotometer spectrophotometer (IMPLEN, München, Germany), ensuring A260/A280 ratios between 1.8 and 2.0. First-strand cDNA synthesis was performed using the 5× All-In-One RT Master Mix kit (Applied Biological Materials Inc., Richmond, BC, Canada). Quantitative Real-Time PCR (qRT-PCR) was performed to assess the expression of genes associated with the Toll signaling pathway in *P. xylostella*. The target genes included: upstream components (*GNBP1*, *Spaetzle*), signal transducers (*Myd88*, *pelle*), and downstream effectors (*Dorsal*, *Defensin*). Elongation factor 1 (EF1) was used as the internal reference gene [30]. Reactions were performed using 2× SYBR Green qPCR Master Mix (EVER-BRIGHT^®^ Inc., Juno Beach, FL, USA) on a CFX96 Touch Real-Time PCR Detection System (Bio-Rad, Hercules, CA, USA). Each 20 µL reaction consisted of 10 µL SYBR Green Mix, 1 µL cDNA, 0.5 µL of each forward and reverse primer (10 µM), and 8 µL of nuclease-free water. The thermal cycling conditions were as follows: 95 °C for 30 s, followed by 40 cycles of 95 °C for 5 s and 60 °C for 30 s. All reactions were performed in triplicate. Relative gene expression levels were calculated using the 2^−ΔΔCt^ method and expressed as mean ± standard error (SE). Primers used for this study are provided in Supplementary Table S1.

### 2.9. Statistical Analysis

All experimental data were expressed as mean ± standard error (SE). One-way ANOVA followed by Duncan’s multiple-range test to evaluate significant differences among four *Tolypocladium* strains treatments. Student’s *t*-test was performed to evaluate significant differences in enzyme activity assays and gene expression level (*p* < 0.05). Mean and ninety lethal times (LT_50_ and LT_90_) and mean and ninety lethal concentrations (LC_50_ and LC_90_) were calculated using Probit analysis. All analyses were performed in SPSS 26.0, and graphs were created with GraphPad Prism 6.0.

## 3. Results

### 3.1. Molecular Identification and Morphological Characterization of Tolypocladium

**Figure 1:**
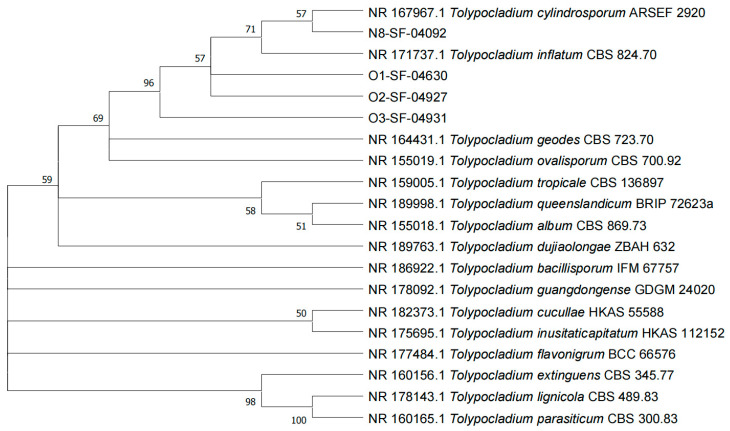
Polylogenetic tree of different *Tolypocladium* strains based on rDNA-ITS sequence. Phylogenetic tree of *Tolypocladium* strains constructed based on rDNA-ITS sequences. The tree was generated using the neighbor-joining method. Bootstrap support values (≥50%) are shown at nodes; values below 50% are omitted for clarity.

The rDNA internal transcribed spacer (ITS) regions of four *Tolypocladium* strains were amplified using universal primers ITS1 and ITS4, yielding single fragments of approximately 500 bp. Sequence analysis showed that strain N8-SF-04092 (581 bp) shared 100% identity with *Tolypocladium cylindrosporum* based on NCBI BLASTn results. Strains O1-SF-04630 (558 bp), O2-SF-04927 (531 bp), and O3-SF-04931 (534 bp) exhibited 98.82%, 100%, and 100% similarity, respectively, with *T. inflatum*. A phylogenetic tree was constructed using representative *Tolypocladium* sequences from GenBank alongside our isolates (Figure 1). Phylogenetic analysis revealed that strain N8-SF-04092 clustered with *T. cylindrosporum* (ARSEF 2920, GeneBank: NR 167967.1), whereas strains O1-SF-04630, O2-SF-04927, and O3-SF-04931 formed a moderately supported clade with *T. inflatum* (CBS 824.70, GeneBank: NR 171731.1).

**Figure 2:**
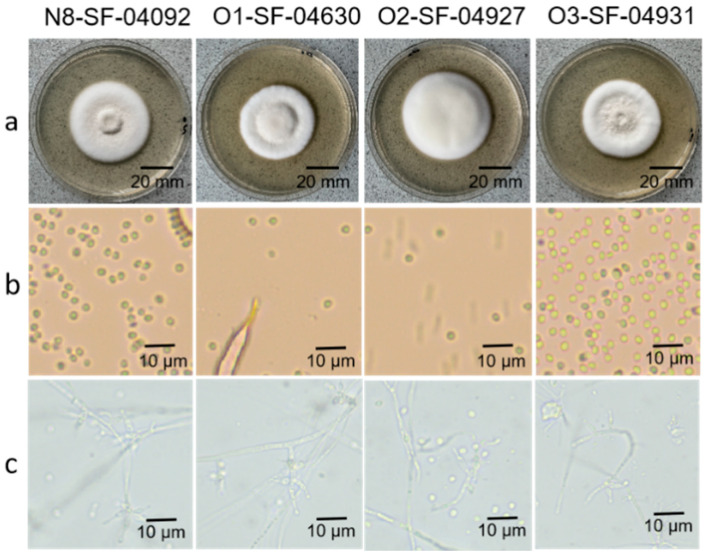
Morphological characteristics of four strains of *Tolypocladium*: (**a**) colony morphology; (**b**) conidia; (**c**) sporulation structure.

After 8 days of incubation on PSA medium at 28 °C, all four *Tolypocladium* strains developed circular, white colonies with distinct morphological traits. Strains N8-SF-04092, O1-SF-04630, and O3-SF-04931 displayed centrally raised colonies with concentric depressions at the periphery and a floccose mycelial texture. In contrast, strain O2-SF-04927 produced evenly elevated colonies characterized by radiating filamentous growth and a velvety appearance. Microscopic examination revealed no notable interspecific differences in conidial morphology. All strains produced small, hyaline conidia that were spherical, sub-spherical, or elliptical, with diameters less than 3 μm (Figure 2). Based on combined morphological characteristics and phylogenetic evidence, strain N8-SF-04092 was identified as *T. cylindrosporum*, while strains O1-SF-04630, O2-SF-04927, and O3-SF-04931 were identified as *T. inflatum*.

### 3.2. Strain Growth and Sporulation Capacity

**Figure 3:**
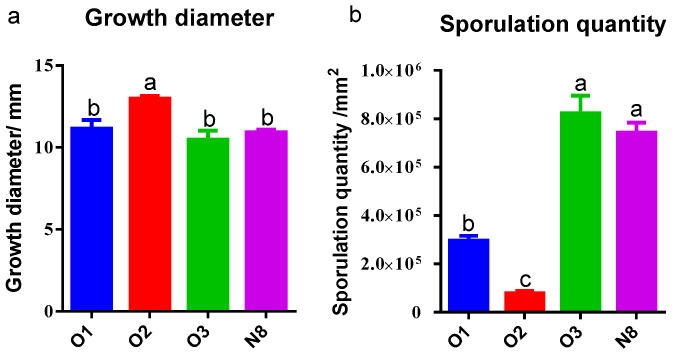
Growth diameter and sporulation quantity of four *Tolypocladium* strains. (**a**) Growth diameter analysis; (**b**) sporulation quantity analysis; O1 represents O1-SF-04630; O2 represents O2-SF-04927; O3 represents O3-SF-04931; N8 represents N8-SF-04092. The same as follows. Error bars represent standard deviations; different letters indicate significant differences (*p* < 0.05).

Growth and sporulation analyses of the four *Tolypocladium* strains revealed significant phenotypic variation (Figure 3). All strains exhibited vigorous growth on a PSA medium, with 7-day corrected colony diameters exceeding 10 mm. Strain O2 showed significantly greater radial growth (12.97 ± 0.27 mm) compared to strains O1, O3, and N8 (*p* < 0.05), whereas no significant differences in growth were observed among O1, O3, and N8 (*p* > 0.05). Sporulation capacity varied markedly among the strains. Strain O3 produced the highest spore density (8.22 ± 1.27) × 10^5^ spores/mm^2^, followed closely by strain N8 (7.43 ± 0.72) × 10^5^ spores/mm^2^, with no significant difference between the two (*p* = 0.399). Both O3 and N8 sporulated significantly more than strains O1 and O2 (*p* < 0.01). Notably, strain O2 exhibited the lowest sporulation capacity, with a spore density of only (7.96 ± 1.59) × 10^4^ spores/mm^2^.

### 3.3. Tolerance of Spores Produced by Toluypocladium *spp.* to Thermal and UV Stress

Thermotolerance assays revealed significant variation in spore germination rates among the four *Tolypocladium* strains following exposure to 45 °C heat shock (Table 1). Strains O1, O3, and N8 exhibited relatively high thermal tolerance, albeit with different response patterns. Spores from strains N8 and O3 maintained stable germination rates throughout the 300 min exposure period compared to the control (0 min) (*p* = 0.12). In contrast, strain O1 showed no significant decline in germination from 0–120 min, but a marked reduction of 41.6% was observed after 300 min of exposure (*p* < 0.05). Strain O2 displayed notable thermo-sensitivity, with a significant drop in germination after just 30 min of heat exposure (*p* < 0.05), and a total reduction of 32.8% after 300 min relative to the control. Overall, the germination rates of N8 and O2 were superior to those of O1 and O3 at all time periods after thermal treatment. At the 120 min timepoint, strains N8 (53 ± 1.7%) and O2 (46.67 ± 0.72%) still showed over 45% gemination rate, which were significantly higher germination rates than O1 and O3 (*p* < 0.05), while O1 and O3 maintained comparably low germination rates (<30%) with no statistical difference between them (*p* > 0.05) (Table 1).

**Figure 4:**
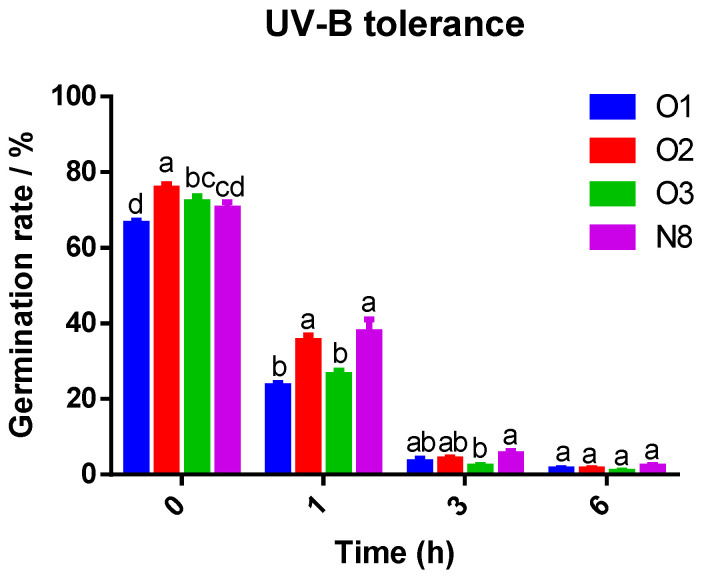
Germination rates of spores from four *Tolypocladium* strains under UV-B stress. Germination rate of four *Tolypocladium* strains after 0, 1, 3, and 6 h irradiation with UV-B. Error bars represent standard deviations; different letters indicate statistically significant differences (*p* < 0.05).

UV-B irradiation significantly inhibited spore germination in a dose-dependent manner across all *Tolypocladium* strains (Figure 4). Under control conditions (0 h), all strains showed high viability (>65% germination), with strain O2 exhibiting the highest germination rate (75.7%). After 1 h of UV-B exposure, all strains experienced a 40–65% reduction in germination compared to the control. Strains O2 (35.77%) and N8 (37.67%) retained significantly higher viability than O1 and O3 (*p* < 0.05), while O1 exhibited the highest sensitivity, with germination dropping to 23.33% (a 64.82% reduction; *p* < 0.05). After 3 h of UV-B exposure, germination rates of all strains declined sharply to below 15.3%, with no significant inter-strain differences observed.

### 3.4. Virulence of Four Tolypocladium Strains Against P. xylostella

Bioassays revealed that all four *Tolypocladium* strains exhibited significant pathogenicity against *P. xylostella* larvae. The earliest larval mortality was observed on day 2 post-inoculation; larvae infected with strain O1 exhibited significantly higher mortality ((16.67 ± 5.93)%) than those treated with other strains (*p* < 0.05) (Table 2). From day 3 to day 7, cumulative mortality progressively increased in all treatment groups. Strains O1, O3, and N8 displayed significantly higher virulence than O2 from day 4 onward (*p* < 0.05), while strain O2 consistently resulted in the lowest larval mortality throughout the assay period. By day 7 post-infection, strain O1 achieved the highest efficacy with a mortality rate of (98.33 ± 1.36)%, followed closely by O3 and N8, both exceeding 90% mortality. All three strains exhibited significantly higher virulence compared to O2 (*p* < 0.05). Mortality in the control group remained significantly lower than all treatments during the entire experimental period (*p* < 0.05). Regression analyses of time–mortality and concentration–mortality responses revealed clear strain-specific differences in virulence (Table 3 and Table 4). Strain O1 had the shortest median lethal time (LT_50_: 3.89 days with 95% fiducial limits (3.44–4.34) days), which was not significantly different from O3 (4.45(4.11–4.89) days) or N8 (4.43(4.03–4.84) days) (*p* > 0.05), but was significantly shorter than that of O2 (*p* < 0.05). Moreover, O1 exhibited the lowest median lethal concentration (LC_50_: 2.79 × 10^4^ spores/mL, with 95% fiducial limits (0.12 × 10^4^–1.36 × 10^5^) spores/mL), indicating its superior biocontrol potential against *P. xylostella*.

### 3.5. Effects of Tolypocladium Infection on Toll Pathway Immune Gene Expression in P. xylostella

**Figure 5:**
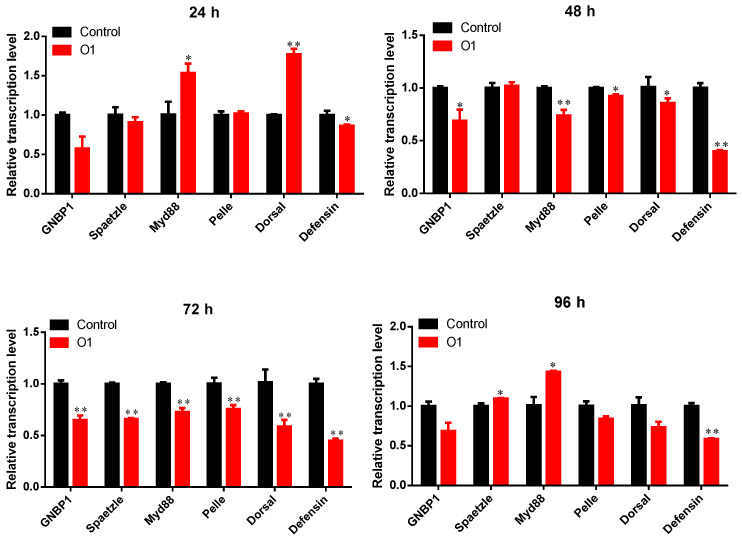
RT-qPCR detection of expression levels of immune-related genes in *P. xylostella* treated by O1-SF-04630. Asterisks indicate significant differences compared to the control group (*p* < 0.05 for *, *p* < 0.01 for **). Error bars represent standard errors. The same notation applies to subsequent figures.

We examined the temporal transcriptional dynamics of Toll pathway-related genes in *P. xylostella* larvae following infection with two highly virulent *Tolypocladium* strains (O1 and N8). Infection with strain O1 induced significant alterations in the expression of key immune genes (Figure 5). Most Toll pathway components were markedly downregulated at 48 h and 72 h post-infection compared to controls (*p* < 0.05), except for *Spaetzle*, which showed no significant change at 48 h (*p* = 0.743). Notably, transient upregulation was observed at specific time points: *Spaetzle* was significantly upregulated at 96 h (*p* < 0.05), *Myd88* was induced at both 24 h and 96 h (*p* < 0.05), and *Dorsal* exhibited strong early activation at 24 h (*p* < 0.01). Despite these upstream activations, the downstream antimicrobial peptide gene *Defensin* was consistently suppressed throughout the infection period (*p* < 0.05).

**Figure 6:**
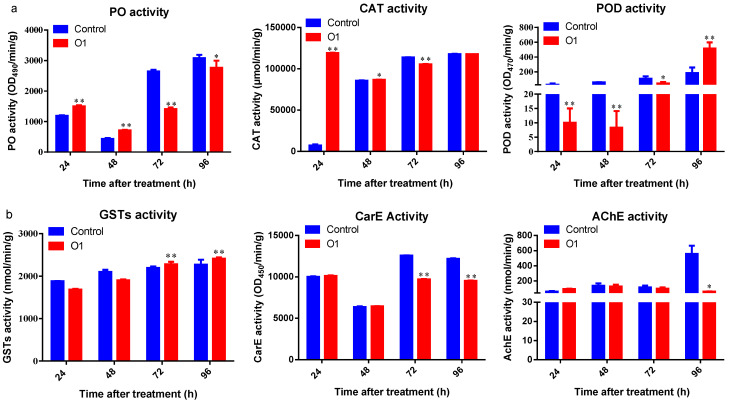
Enzyme activity profiles in *P. xylostella* larvae following infection with *Tolypocladium inflatum* strain O1-SF-04630. (**a**) Temporal changes in protective enzyme activities (PO, CAT, POD) at 24, 48, 72, and 96 h post-treatment. (**b**) Temporal changes in detoxification enzyme activities (AChE, CarE, GSTs) at 24, 48, 72, and 96 h post-treatment.

Following infection with strain O1, *P. xylostella* larvae exhibited significant fluctuations in protective enzyme activities (Figure 6). Phenol oxidase (PO) and catalase (CAT) activities followed a similar biphasic pattern—both enzymes were significantly upregulated at 24 h and 48 h post-treatment (*p* < 0.05) but subsequently inhibited at 72 h and 96 h (*p* < 0.05) compared to controls. In contrast, peroxidase (POD) activity was significantly suppressed at 24 h, 48 h, and 72 h (*p* < 0.05) but showed a marked increase at 96 h post-treatment (*p* < 0.01). In terms of detoxifying enzymes, significant changes were observed after 72 h of exposure. Glutathione S-transferase (GST) activity remained comparable to controls at 24 h and 48 h but was significantly elevated at 72 h and 96 h (*p* < 0.01). Carboxylesterase (CarE) activity declined substantially after 72 h and was significantly lower than control levels at both 72 h and 96 h (*p* < 0.01). Acetylcholinesterase (AChE) activity was significantly suppressed only at 96 h post-infection (*p* < 0.05), with no significant differences observed at earlier time points.

**Figure 7:**
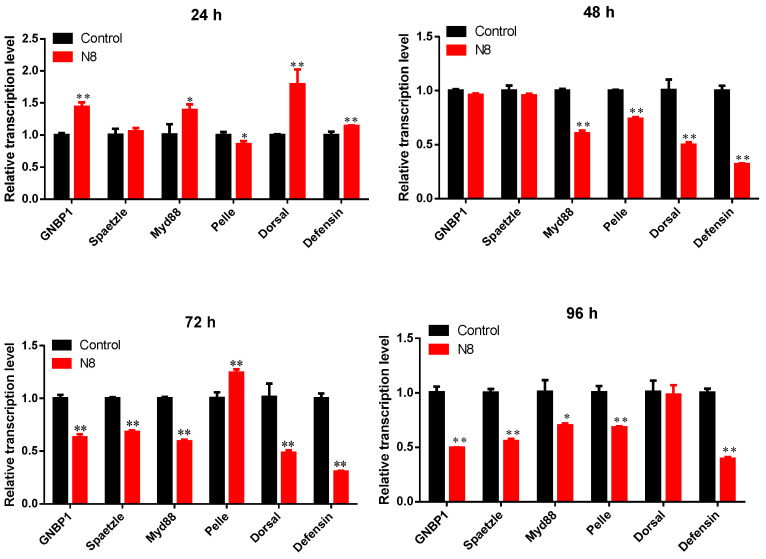
RT-qPCR analysis of immune-related gene expression in *P. xylostella* larvae following infection with *Tolypocladium cylindrosporum* strain N8-SF-04092.

Following exposure to strain N8, *P. xylostella* larvae exhibited time-dependent modulation of Toll pathway-related gene expression (Figure 7). At 24 h post-treatment, the upstream genes *GNBP1* and *Spaetzle* were activated, with *GNBP1* expression significantly higher than the control (*p* < 0.01). The membrane signaling factor *Myd88* also showed significant upregulation (*p* < 0.05), and the transcription factor *Dorsol* was activated (*p* < 0.01), leading to a significant accumulation of the antimicrobial peptide Defensin (*p* < 0.01). After 48 h, 72 h, and 96 h of treatment, the expression levels of all genes were inhibited to varying degrees, with only *Pelle* showing a significant upregulation at 72 h (*p* < 0.01).

**Figure 8:**
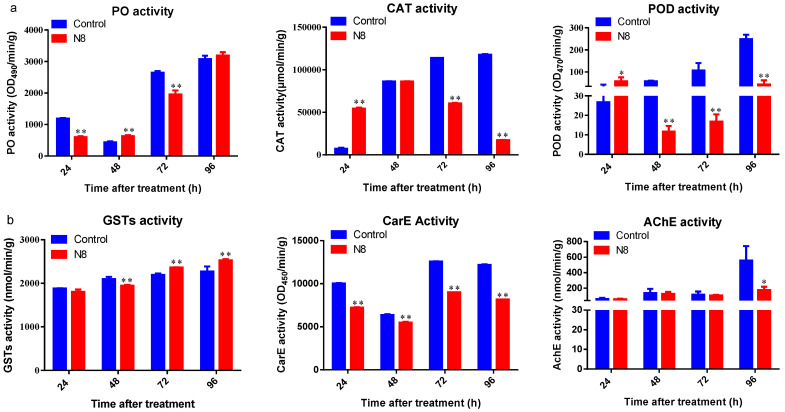
Enzymatic activity changes in *P. xylostella* larvae following treatment with *Tolypocladium cylindrosporum* strain N8-SF-04092. (**a**) Temporal variation in protective enzyme activities (PO, CAT, POD) at 24 h, 48 h, 72 h, and 96 h post-infection. (**b**) Temporal variation in detoxification enzyme activities (CarE, AChE, GSTs) at corresponding time points.

Following treatment with strain N8, phenol oxidase (PO) activity in *P. xylostella* larvae exhibited a biphasic trend, characterized by an initial increase followed by suppression (Figure 8a). PO activity was significantly suppressed at 24 h and 72 h post-treatment (*p* < 0.01), while a marked upregulation was observed at 48 h compared to the control group (*p* < 0.01). Catalase (CAT) and peroxidase (POD) activities followed a similar pattern, with significant activation at 24 h (*p* < 0.05), followed by a progressive decline at 48 h, 72 h, and 96 h. The strongest inhibition for both enzymes was recorded at 72 h (*p* < 0.02).

Glutathione S-transferase (GST) activity also demonstrated a biphasic response (Figure 8b). A significant reduction was observed at 48 h (*p* < 0.01), followed by a substantial increase at 72 h and 96 h (*p* < 0.01). In contrast, carboxylesterase (CarE) and acetylcholinesterase (AChE) activities showed a consistent inhibitory trend. CarE activity was significantly reduced at all time points (24 h, 48 h, 72 h, and 96 h; *p* < 0.01), while AChE activity showed a significant decrease only at 96 h post-treatment (*p* < 0.05), with no significant differences at earlier time points.

## 4. Discussion

*Plutella xylostella*, a globally destructive pest of cruciferous crops, has evolved resistance to over 50 insecticidal compounds, including synthetic and biological formulations such as *Bacillus thuringiensis* (Bt) [31,32,33]. The widespread and rapid development of resistance necessitates alternative control strategies. Entomopathogenic fungi represent promising biocontrol agents due to their ecological compatibility, capacity for cuticular penetration, and persistent insecticidal effects. Common fungal agents reported for the control of *P. xylostella* include *Beauveria bassiana* [34], *Metarhizium pingshaense* [35], *Paecilomyces cicadae* [36], and *Zoophthora radicans* [37]. However, the entomopathogenic potential of *Tolypocladium* species against this pest has remained unexplored until now.

In the present study, we identified four *Tolypocladium* strains exhibiting high virulence against *P. xylostella* larvae. Strain O1-SF-04630 showed the highest pathogenicity, achieving a cumulative larval mortality of 98.3% within seven days and an LT_50_ of 3.89 ± 0.45 days. Strain N8-SF-04092, though marginally less virulent, exhibited superior mycelial growth and sporulation. These complementary attributes highlight the potential of both strains for development as microbial control agents.

Members of the genus *Tolypocladium* are ecologically diverse and globally distributed, producing a wide range of bioactive compounds with antifungal, antitumor, and insecticidal properties. Among the 43 known species (International Mycological Directory, 2020 [38]), *T. inflatum* is notable for producing cyclosporins—clinically important immunosuppressants [15,39]. Other secondary metabolites such as ophiocordin, efrapeptins, aphidicolin, and chlamydosporol also exhibit potent bioactivities [40]. Notably, other bioactive metabolites derived from this genus—including ophiocordin (a potent protease inhibitor), efrapeptins (mitochondrial ATPase inhibitors), aphidicolin (a DNA replication inhibitor), and chlamydosporol—have also been systematically investigated [41,42,43]. However, despite this chemical richness, studies on the direct insecticidal effects of *Tolypocladium* strains remain scarce.

Our data indicate that *T. inflatum* strain O1-SF-04630 is highly effective against *P. xylostella*, whereas *T. cylindrosporum* strain N8-SF-04092 demonstrated substantial virulence, with 96.7% cumulative mortality and an LT_50_ only 0.54 days longer than O1. These findings are consistent with previous reports of *T. cylindrosporum* pathogenicity in other insects such as *Delia radicum* and *Aedes aegypti* [44,45]. Additionally, strain N8 exhibited superior growth and sporulation, suggesting potential for large-scale production.

Ultraviolet radiation and thermal stress are two major abiotic factors that significantly limit the development and field efficacy of entomopathogenic fungi in biological pest control applications. Among these, UV-B radiation has been shown to exert strong inhibitory effects on fungal viability and development. For instance, a 15% increase in UV-B irradiance can lead to a reduction in conidial germination rates by up to 40% in UV-sensitive fungal species [46]. Thermal tolerance also plays a critical role in fungal survival under field conditions. While dormant conidia of *Metarhizium robertsii* can endure 50 °C for 24 h, vegetative conidia lose viability after only 4 h at 45 °C [47].

In this study, all four *Tolypocladium* strains exhibited relatively high tolerance to 45 °C wet heat stress. Notably, after 120 min of exposure, strains N8 and O2 showed significantly higher germination rates—53% and 46.67%, respectively, compared to strains O1 and O3, which both exhibited rates below 30%. Remarkably, strain N8-SF-04092 retained a germination rate of 48.33% even after 300 min of heat stress at 45 °C, a value not significantly different from the control group (0 min; *p* = 0.12). These findings align with the results of Santos et al. (2011), who reported that *T. cylindrosporum* strains (ASEF 3392, 5558) maintained >40% germination following 4 h of humid heat stress at 45 °C, outperforming *M. robertsii* (ARSEF 2575) [48]. Collectively, these data suggest that under certain environmental conditions, *Tolypocladium* species may serve as viable alternatives to more heat-sensitive entomopathogenic fungi.

During host infection, entomopathogenic fungi elicit a complex immune response from the insect host [49,50]. The innate immune system of insects is composed of two primary arms: cellular and humoral immunity. Cellular immunity is mediated by hemocytes and involves mechanisms such as phagocytosis, encapsulation, and nodulation [51]. In contrast, humoral immunity is driven by systemic effectors, including antimicrobial peptides (AMPs), phenoloxidase (PO), and pattern recognition receptors (PRRs) [52]. Upon fungal invasion, PRRs such as GNBP and βGRP detect pathogen-associated molecular patterns (PAMPs) like β-1,3-glucans and lipopolysaccharides. This recognition initiates a serine protease cascade involving PPAE and PAP, ultimately cleaving prophenoloxidase (PPO) into active PO, which oxidizes phenolic substrates to form quinones and, subsequently, melanin [53,54]. This melanization process is a rapid immune response and is often completed within minutes [55].

In the present study, PO activity was significantly induced at 24 h and 48 h post-infection by the O1 strain and similarly elevated at 48 h following N8 infection (*p* < 0.05). However, PO activity decreased markedly at 72 h in both treatments (Figure 6a and Figure 8a). This trend is similar to the PO activity pattern observed in *P. xylostella* treated with *M. anisopliae*, where PO activity peaked between 24 and 48 h and then gradually declined at 72 and 96 h [56], suggesting that PO-mediated defense plays a pivotal role in early stages of infection but becomes suppressed as the fungus establishes systemic infection. This decline likely reflects a pathogenic mechanism of *Tolypocladium* similar to that of other entomopathogenic fungi involving immune suppression. Although the absence of a positive control limits direct efficacy comparisons, the consistent dose–response patterns and enzyme activity trends support the biological activity of *Tolypocladium* strain O1 and N8. Future studies should include standardized controls (e.g., commercial biopesticides like Metarhizium) to validate these findings.

The Toll signaling pathway is central to the antifungal immune response in insects, primarily through regulation of AMPs. Our gene expression analyses revealed distinct modulation patterns of Toll pathway components in response to fungal infection. Strain O1 induced early (24 h) and late (96 h) upregulation of select Toll pathway genes, but this was accompanied by significant downregulation of *Defensin* across all time points (*p* < 0.05; Figure 7), indicating an overall suppressive effect. Strain N8 elicited transient activation of the pathway at 24 h, but this was followed by broad suppression between 48 and 96 h, mirroring the dynamics of PO activity. These results suggest that *Tolypocladium* infection initially activates host immunity but progressively inhibits immune signaling, facilitating fungal colonization and pathogenesis.

Catalase (CAT) and peroxidase (POD) are essential antioxidant enzymes that mitigate oxidative damage caused by pathogen-induced reactive oxygen species (ROS). These enzymes convert hydrogen peroxide into water and oxygen, preventing cellular damage and maintaining redox homeostasis [55]. Our data showed a biphasic pattern in antioxidant enzyme activity following fungal challenge. Specifically, CAT activity in O1-infected larvae and both CAT and POD activities in N8-treated larvae peaked at 24 h (*p* < 0.05) before declining significantly after 72 h (Figure 6a and Figure 8a). Similar biphasic responses have been documented in *P. xylostella* infected with *M. anisopliae* (Ma1), showing increased levels of free radical scavenging enzymes such as CAT and POD, between 48 and 96 h compared to the control, followed by a steady decline in protective enzyme activity after 96 h [57]. A comparable pattern was also observed in larvae of *Dendrolimus tabulaeformis* and *Apriona germari* with *Beauveria bassiana*, where antioxidant enzyme activity initially increases but was subsequently downregulated during later stages of infection [58,59]. These findings imply that an early oxidative stress response is mounted by the host, but sustained fungal infection suppresses antioxidant defenses, thereby exacerbating cellular damage and contributing to mortality.

In addition to protective enzymes, insects utilize detoxification enzymes such as carboxylesterase (CarE), glutathione S-transferase (GST), and acetylcholinesterase (AChE) to neutralize fungal toxins and preserve physiological balance. Our study showed delayed responses of these enzymes. CarE and AChE activities remained largely unchanged or were significantly suppressed across infection timelines following both O1 and N8 treatments, while GST activity was notably upregulated at 72–96 h in both treatment groups (Figure 6b and Figure 8b). GSTs are pivotal in detoxifying xenobiotic and endogenous toxic compounds by catalyzing their conjugation with glutathione. Furthermore, they play a crucial role in scavenging oxidative byproducts during tissue injury. The upregulation of GSTs at later infection stages likely reflects a compensatory response to increasing tissue damage and toxin accumulation [60].

By integrating immune gene expression profiles and enzyme activity dynamics, it can be inferred that *P. xylostella* mounts an immediate defense response following *Tolypocladium* infection, as evidenced by the activation of PO, POD, CAT, and Toll pathway genes. However, as infection progresses, these defense mechanisms are progressively suppressed, allowing fungal proliferation and the accumulation of toxic metabolites. This collapse in immune and metabolic homeostasis ultimately leads to larval death. These results provide critical insights into the mechanisms of fungal virulence and host defense modulation, further supporting the potential of *Tolypocladium* species as effective biocontrol agents against *P. xylostella*.

## 5. Conclusions

In this study, all four tested *Tolypocladium* strains exhibited considerable pathogenicity against the *P. xylostella*. Notably, *T. inflatum* O1-SF-04630 demonstrated the highest virulence, while *T. cylindrosporum* N8-SF-04092 showed superior sporulation capacity and remarkable tolerance to both UV radiation and high-temperature stress. These two elite strains achieved pathogenic infection primarily through suppressing host immune enzyme activities and downregulating defense-related gene expression, indicating significant potential for biocontrol applications.

## Figures and Tables

**Table 1:** Germination rates of spores of four *Tolypocladium* strains under different durations of heat treatment at 45 °C.

45 °C Heat Stress	Germinate Rates %			
Time (min)	N8-SF-04092	O1-SF-04630	O2-SF-04927	O3-SF-04931
0	(56.67 ± 1.96) aA	(25.67 ± 0.98) aC	(63 ± 1.89) aA	(32.33 ± 1.52) aB
10	(54.33 ± 1.44) aA	(23.33 ± 2.33) aB	(53.33 ± 1.19) bA	(26.67 ± 0.27) aB
30	(52.33 ± 3.07) aB	(23 ± 0.47) aC	(62.33 ± 1.19) aA	(29.33 ± 1.19) aC
60	(54.67 ± 2.23) aA	(22.67 ± 0.54) aC	(42.33 ± 1.19) cB	(28 ± 0.94) aC
120	(53 ± 1.7) aA	(23 ± 0.94) aC	(46.67 ± 0.72) cB	(27.67 ± 1.44) aC
300	(48.33 ± 2.88) aA	(15 ± 0.94) bC	(46 ± 2.62) dA	(27 ± 2.94) aB

Results are presented as mean ± standard errors (SE). One-way ANOVA was performed using Duncan’s multiple-range test. Lowercase letters within the same column indicate significant differences (*p* < 0.05) among different thermal times under the same strains. Uppercase letters within the same row indicate significant differences (*p* < 0.05) among different strains under the same thermal time.

**Table 2:** Analysis of the significance of differences in cumulative mortality among four strains.

Treatment	Days After Treatment (Day)						
Spores/mL	1	2	3	4	5	6	7
N8-SF-04092	(1.67 ± 1.36)% a	(3.33 ± 1.36)% b	(11.67 ± 2.72)% b	(40 ± 4.08)% ab	(70 ± 6.24)% a	(85 ± 6.24)% a	(96.67 ± 2.72)% a
O1-SF-04630	(3.33 ± 2.72)% a	(16.67 ± 5.93)% a	(28.33 ± 4.91)% a	(51.67 ± 3.6)% a	(71.67 ± 1.36)% a	(90 ± 2.36)% a	(98.33 ± 1.36)% a
O2-SF-04927	(0 ± 0)% a	(1.67 ± 1.36)% b	(3.33 ± 2.72)% b	(20 ± 0)% c	(40 ± 2.36)% b	(60 ± 4.08)% b	(70 ± 4.71)% b
O3-SF-04931	(0 ± 0)% a	(1.67 ± 1.36)% b	(11.67 ± 1.36)% b	(36.67 ± 4.91)% b	(71.67 ± 7.2)% a	(88.33 ± 3.6)% a	(93.33 ± 3.6)% a
Control	(0 ± 0)% a	(0 ± 0)% b	(0 ± 0)% b	(0 ± 0)% d	(1.67 ± 1.36)% c	(1.67 ± 1.36)% c	(3.33 ± 1.36)% c

Note: Results are presented as mean ± standard errors (SE). One-way ANOVA was performed using Duncan’s multiple-range test. Data in the same column followed by different lowercase letters represent a significant difference at the 0.05 level.

**Table 3:** Mean (LT_50_) and 90 (LT_90_) lethal time of four strains to third-instar larvae of *P. xylostella* at a concentration of 1 × 10^8^ spores mL^−1^.

Treatment Strain	LT_50_ (days) (95% Fiducial Limits)	LT_90_ (days) (95% Fiducial Limits)	Probit Equation	*X* ^2^	*p* Value
O1	3.89 (3.44–4.34) a	6.03 (5.43–6.97) a	y = −2.336 + 0.6x	0.556	0.990
O2	5.71 (5.23–6.34) b	8.01 (7.18–9.55) b	y = −3.183 + 0.557x	1.4	0.924
O3	4.45 (4.11–4.89) a	6.19 (5.69–6.96) a	y = −3.416 + 0.759x	1.716	0.887
N8	4.43 (4.03–4.84) a	6.21 (5.68–7.03) a	y = −3.202 + 0.723x	0.703	0.983

Different letters indicate significant differences according to the overlap of the fiducial limits.

**Table 4:** Mean (LC_50_) and 90 (LT_90_) lethal concentration of each strain on the 7th day of treatment to third-instar larvae of *P. xylostella*.

Treatment Strain	LC_50_ (Spores mL^−1^) (95% Fiducial Limits)	LC_90_ (Spores mL^−1^) (95% Fiducial Limits)	Probit Equation	*X* ^2^	*p* Value
O1	2.79 × 10^4^ (0.12 × 10^4^–1.36 × 10^5^) a	3.31 × 10^8^ (0.11 × 10^8^–4.65 × 108^15^) a	y = −2.94 + 4.537x	4.45	0.217
O2	1.43 × 10^5^ (7.24 × 10^2^–1.86 × 10^9^) a	2.98 × 10^21^ (1.86 × 10^10^–9.55 × 10^44^) a	y = −1.473 + 2.068x	0.491	0.921
O3	4.37 × 10^4^ (0.09 × 10^4^–2.74 × 10^5^) a	1.08 × 10^10^ (0.01 × 10^10^–1.69 × 10^30^) a	y = −2.55 + 3.826x	3.567	0.312
N8	5.58 × 10^4^ (0.34 × 10^4^–2.74 × 10^5^) a	1.37 × 10^9^ (0.03 × 10^9^–5.87 × 10^17^) a	y = −3.048 + 4.506x	4.826	0.185

Different letters indicate significant differences according to the overlap of the fiducial limits. Note: The spore suspension of 1 × 10^8^ spores/mL was diluted in a 1:9 ratio to 10^7^, 10^6^, 10^5^, and 10^4^ spores/mL for each treatment group (n = 20 insects per concentration, three biological repetitions).

## Data Availability

The original contributions presented in this study are included in the article/Supplementary Material. Further inquiries can be directed to the corresponding author.

## Supplementary Materials

The following supporting information can be downloaded at https://www.mdpi.com/article/10.3390/insects16080859/s1, Table S1: Primers for RT-qPCR.
